# Mad Dogs

**Published:** 1879-08

**Authors:** 


### MAD DOGS.

The only safe plan is to regard as mad any dog that bites. Persons have died of hydrophobia from being bitten by dogs that have lived for years afterward. Until we know more of the nature of this fearful disease there will be no safety in trusting to our judgment as to whether a dog is mad or not; hence every such wound should be promptly treated. Wash it with soap and water, squeeze the parts to make the wound bleed as much as possible. Make a cupping glass by putting a small piece of burning paper into a tumbler or tea-cup and placing it over the wound while the paper is yet blazing. This will draw out some blood and probably the poison with it. But still it is not well to stop here. Get some carbolic acid or nitric acid, and with a feather or camel's-hair brush, or even a stick, dipped into the acid, touch the entire raw surface of the wound with it. A red-hot iron has been used for this purpose and is really less painful, if quickly touched, than acids. Cutting out the wounded part instantly, when that can be done without permanent injury, is a radical cure, but I do not think it necessary.

The above treatment is severe, but it is nothing in comparison to hydrophobia, or even to the fear of the disease to torture one for a life-time after having been bitten.

More cases of hydrophobia result from slight scratches with the teeth of dogs than from severe bites, because the latter bled freely and wash away the poison.

Among the recent cures for hydrophobia the inhalation of three cubic feet of oxygen just before each paroxysm is said to have been most successful.

